# An analysis of the health status of the United Arab Emirates: the ‘Big 4’ public health issues

**DOI:** 10.3402/gha.v6i0.20100

**Published:** 2013-02-05

**Authors:** Tom Loney, Tar-Ching Aw, Daniel G. Handysides, Raghib Ali, Iain Blair, Michal Grivna, Syed M. Shah, Mohamud Sheek-Hussein, Mohamed El-Sadig, Amer A. Sharif, Yusra El-Obaid

**Affiliations:** 1 Institute of Public Health, College of Medicine and Health Sciences, United Arab Emirates University, Al Ain, United Arab Emirates; 2 School of Public Health, Loma Linda University, California, United States; 3 Dubai Health Authority, Dubai, United Arab Emirates

**Keywords:** globalisation of public health, non-communicable disease, United Arab Emirates

## Abstract

**Background:**

The United Arab Emirates (UAE) is a rapidly developing country composed of a multinational population with varying educational backgrounds, religious beliefs, and cultural practices, which pose a challenge for population-based public health strategies. A number of public health issues significantly contribute to morbidity and mortality in the UAE. This article summarises the findings of a panel of medical and public health specialists from UAE University and various government health agencies commissioned to report on the health status of the UAE population.

**Methods:**

A systematic literature search was conducted to retrieve peer-reviewed articles on health in the UAE, and unpublished data were provided by government health authorities and local hospitals.

**Results:**

The panel reviewed and evaluated all available evidence to list and rank (1=highest priority) the top four main public health issues: 1) Cardiovascular disease accounted for more than 25% of deaths in 2010; 2) Injury caused 17% of mortality for all age groups in 2010; 3) Cancer accounted for 10% of all deaths in 2010, and the incidence of all cancers is projected to double by 2020; and 4) Respiratory disorders were the second most common non-fatal condition in 2010.

**Conclusion:**

The major public health challenges posed by certain personal (e.g. ethnicity, family history), lifestyle, occupational, and environmental factors associated with the development of chronic disease are not isolated to the UAE; rather, they form part of a global health problem, which requires international collaboration and action. Future research should focus on population-based public health interventions that target the factors associated with the development of various chronic diseases.

The United Arab Emirates (UAE) is a country composed of seven emirates (Abu Dhabi, Ajman, Dubai, Fujairah, Ras Al Khaimah, Sharjah, and Umm Al-Quwain), formed in 1971, and is located in the southeast of the Arabian Peninsula (1). Since the discovery of oil, the UAE has experienced significant economic and industrial growth, particularly in the petroleum, aviation, maritime, construction, and health care industries (2). Several mega-projects exemplify the industrial progress over the past 10 years, including the construction of the world's tallest building (Burj Khalifa) and largest shopping complex (Dubai Mall), Jebel Ali Port and Free Zone, Dubai International Airport, and numerous artificial islands: Yas Island, Palm Dubai, and a man-made archipelago called The World. In addition, the UAE has an expanding manufacturing base with aluminium, steel, iron, and textiles contributing significantly to exports.

## Population growth and demographics of the UAE

Population growth is the product of natural growth (births minus deaths) and growth from net migration (3). Migrant workers are recruited from all over the world to satisfy the manpower demands of the fast-paced economic and industrial developments in the UAE (4). Consequently, the UAE population has increased substantially over the past four decades, and this is primarily due to the high net inward migration of expatriate workers (population estimates: 287,000 in 1971, 4.1 million in 2005, 8.3 million in 2010) (5). Indeed, mass recruitment of migrant workers has created an unusual population structure, with the total UAE population composed of approximately 11% (950,000) Emiratis, and the rest expatriates of varying nationalities (6). Similarly, the total population of Abu Dhabi (the largest emirate in the UAE) is estimated to be 2.3 million, with over half of the population being expatriate males aged 20–59 years (6).

As a result of expatriate workforce recruitment for industrial projects, males outnumber females 3:1 in the overall UAE population (nationals and non-nationals); however, there are approximately equal numbers of male and female UAE nationals (3). Among non-nationals, the ratio of males to females is 3.7:1 due to the imbalance between the number of expatriate males employed in construction compared with migrant females working in hospitality, health care, or domestic service (3). Principally, there is an apparent distribution of migrant workers by nationality; construction workers and manual labourers tend to be from the Indian subcontinent; middle managers and health care workers from the Philippines, India, and neighbouring Arab countries; and senior management and consultants from the UAE, Europe, North America, and Australasia. As such, the UAE is composed of a multinational population, with varying educational backgrounds, religious beliefs, and cultural practices, which pose a challenge for population-based public health strategies.

The primary aim of this article was to utilise secondary data from existing peer-reviewed journal publications and reports of government agencies and related health organisations to comment on the key public health issues in the UAE.

## Materials and methods

The following method was used to obtain secondary data from existing peer-reviewed journal articles and reports of government agencies and related health organisations:

1. systematic search of the published literature, using defined keywords;
2. through personal contact with senior health officers at health authorities, government agencies, and local hospitals; and
3. from publications/reports by health authorities, government agencies, and local hospitals.

### Literature search strategy

A systematic literature search was conducted to retrieve peer-reviewed scientific and medical journal articles on health in the UAE. Electronic databases (MEDLINE [accessed by PubMed], EMBASE, PsycINFO) were searched covering the period from 1950 to January 2012, utilising a combination of the following MESH terms, free-text words, and entry terms – ‘health, public health, morbidity, mortality, diabetes, overweight, obesity, population, United Arab Emirates’ (see Supplementary Material File 1 for an example of search syntax used for PubMed). The literature search was conducted in accordance with the Preferred Reporting Items for Systematic Reviews and Meta-Analyses (PRISMA) guidelines (7), which stipulate the components (e.g. multiple data sources, unpublished) associated with a high-sensitivity search strategy. In addition, references of published studies were searched manually for pertinent articles and national/local health authorities (e.g. Health Authority Abu Dhabi) were contacted for annual statistics and data. Sources of unpublished data included personal communication with senior health officers at the Health Authorities of Dubai and Abu Dhabi, the UAE Ministry of Health, publications produced by local hospitals (e.g. the cancer registry of Tawam hospital in Al-Ain, Abu Dhabi), and in-house data collected from various on-going faculty, staff, and student research projects.

#### Eligibility criteria

Eligible studies included only empirical research papers that were relevant to the health status of the UAE. Exclusion criteria were:

1. studies that highlighted the UAE in the abstract but subsequently pooled data to produce estimates for the Gulf Region or the Gulf Cooperation Council (GCC) states (i.e. Bahrain, Kuwait, Oman, Qatar, Saudi Arabia, and UAE);
2. articles that were not available in English; and
3. duplicate publications or sub-studies of included articles. The systematic literature search yielded a total of 185 citations and 179 abstracts. Table 1 summarises the findings from the literature retrieval.

**Table 1:** Summary of citations and abstracts retrieved from literature search

	Citations	Abstracts
Overall	185	179
Mortality	6	5
Paediatric	2	2
Adult (Female/maternal)	2	1
Environmental	2	2
		
UAE general health	6	5
Primary health care	1	1
Public health	3	2
Labour migration and expatriates	2	2
		
Injury	16	16
Traffic	8	8
Paediatric	3	3
Occupational	3	3
Older adult	1	1
Animal (Camel)	1	1
		
Neonatal and infant health	8	8
Nutrition and vitamin deficiency	3	3
Congenital anomalies	2	2
Birth weight and growth	3	3
		
Older adult health	2	2
Institutionalised	1	1
Community	1	1
		
Genetic health	14	14
UAE nationals	3	3
Consanguinity	3	3
Infant/birth defect	2	2
Blood/hepatitis	6	6
		
Cardiovascular health	12	12
Adult	12	12
UAE	4	4
Abu Dhabi	2	2
Al Ain	3	3
Dubai and Northern Emirates	1	1
Sharjah	2	2
		
Diabetes	13	13
Adult	6	6
UAE nationals only	3	3
Complications and cost	5	5
Paediatric	2	2
		
Metabolic syndrome	5	5
Adult	4	4
Paediatric	1	1
Overweight and obesity	15	15
Adult	6	6
Paediatric	9	9
		
Cancer	8	7
Adult	5	4
Paediatric	3	3
		
Chronic disease and disorders	7	6
Multiple sclerosis	1	1
Anorectal disease	1	0
Arthritis	1	1
Renal disease	1	1
Dermatitis and allergies	3	3
		
Respiratory disease and disorders	6	6
Adult	1	1
Paediatric	3	3
Both	2	2
		
Blood disorders	5	5
Adult	1	1
Paediatric	3	3
Both	1	1
		
Infectious diseases	8	7
Intestinal parasites and bacteria	2	2
Meningitis	2	2
Hepatitis (A and C)	2	1
Tuberculosis	1	1
Antibiotic use	1	1
		
Oral health	7	6
Adult	1	1
Paediatric	6	5
		
Smoking behaviour	6	6
Adult (health professionals)	4	4
Paediatric	2	2
		
Physical activity and exercise	3	3
Adult	1	1
Paediatric	2	2
		
Mental health	21	21
Adult	11	11
Al Ain	5	5
Dubai	4	4
Sharjah	2	2
Paediatric	9	9
Older Adult	1	1
		
Occupational health	17	17
Noise and heat	6	6
Heavy metal exposure	3	3
Particulates and exhaust fumes	4	4
Pesticides	4	4

#### Data extraction and synthesis

Phase one of data extraction and synthesis (DEaS) entailed one investigator (who was not blinded to authors, institutions, or journals) reviewing and evaluating the titles and abstracts of retrieved articles. Abstracts that did not provide enough information about whether the publication contained suitable and relevant information about the health status of the UAE were retrieved for full text evaluation. Phase two of DEaS involved collating the research articles into 20 specific health topics (presented in Table 1; see Supplementary Material File 2 for a full reference list of journal articles reviewed). Phase three of DEaS required a panel of medical and public health specialists from the College of Medicine and Health Sciences, UAE University and government health agencies to convene and review the evidence for the 20 specific health topics and select the four priority public health areas for the UAE. This phase employed an iterative and inductive process, which involved the panel reviewing, discussing, and re-reviewing the evidence for each health topic until consensus was reached on the four most important public health issues for the UAE.

Phase four of DEaS used a review and rank exercise, which involved the panel evaluating the available evidence for each of the four public health areas and ranking them in order of priority (1=highest priority). The four selected public health priority areas were:

1. cardiovascular disease;
2. injury (including road traffic, child, and occupational injuries);
3. cancers; and
4. respiratory disorders

Following the review and rank exercise, medical and public health experts from each of the four identified public health priority areas were invited to produce a brief statement on the conditions in relation to the current health status of the UAE population, and to produce recommendations for improving health.

## Results

### Key health indicators of the UAE

#### Fertility & life expectancy in the UAE

Fertility rates, the predominant driver of population growth for nationals, have declined over the past 30 years with the total fertility rate (i.e. average number of children that would be born to a woman over her lifetime) decreasing from 4.4 to 2.4 per woman between 1990 and 2010 (3). A decline in birth rates has been attributed to urbanisation, changing attitudes about family size, and improved education and work opportunities for women resulting in delayed marriage. Life expectancy in the UAE continues to improve slowly, and the most recent estimates from 2009 report 77 and 79 years for males and females, respectively (8).

### Communicable diseases in the UAE

In view of the high inward migration of expatriate workers and transient influx of tourists from all over the world, infectious diseases remain an important area for public health in the UAE. However, communicable diseases were not included in the top four public health priority areas, as the UAE is considered a high-income country (based on global economic indicators) that is deemed to have passed through the epidemiologic transition (3). As such, the burden of infectious and parasitic diseases in the UAE is low due to improvements in the standard of living and both the quality and availability of health care services. To prevent the re-emergence or outbreaks of infectious disease, the UAE has invested significant resources into population-based control measures, such as immunisation, surveillance, and the availability of post-exposure treatment (where possible). One major public health achievement in the UAE is the success rate of the national immunisation program for children under the age of 5 years. Due to the high uptake of immunisation, the rates of childhood communicable diseases in the UAE are very low. In addition, all expatriate workers seeking employment in the UAE are screened for communicable diseases, such as tuberculosis (by chest X-ray) and human immunodeficiency virus (by serology), before acquiring residence status. Additional screening for other communicable diseases is required for certain occupational categories, such as healthcare workers, cooks, house maids, and drivers.

### Public health priority area 1: cardiovascular disease

Since 1971, the UAE has experienced tremendous economic and industrial development, resulting in an increase in the affluence of the Emirati population (9) and a shift from a traditional semi-nomadic lifestyle to a modern, urbanised, and technology-driven lifestyle characterised by reduced occupational, domestic, and leisure-time physical activity, coupled with the overconsumption of energy-dense convenience foods with poor nutritional content. Consequently, there has been a dramatic increase in the prevalence of obesity, diabetes, and cardiovascular disease. The World Health Organisation estimates that the burden of chronic diseases is rapidly increasing worldwide, with the largest increase in cardiovascular disease occurring in the Eastern Mediterranean Region. Indeed, the UAE has one of the highest age-standardised death rates for cardiovascular disease in the world, that is, 308.9 per 100,000 for males and 203.9 per 100,000 for females. Recent mortality statistics for the emirate of Abu Dhabi in 2010 reveal that 29% of all deaths were due to cardiovascular disease (10). In the absence of any major changes in lifestyle risk factors at a population level, these rates are set to increase further as the youthful population ages.

### Public health priority area 2: injury

**Fig. 1:**
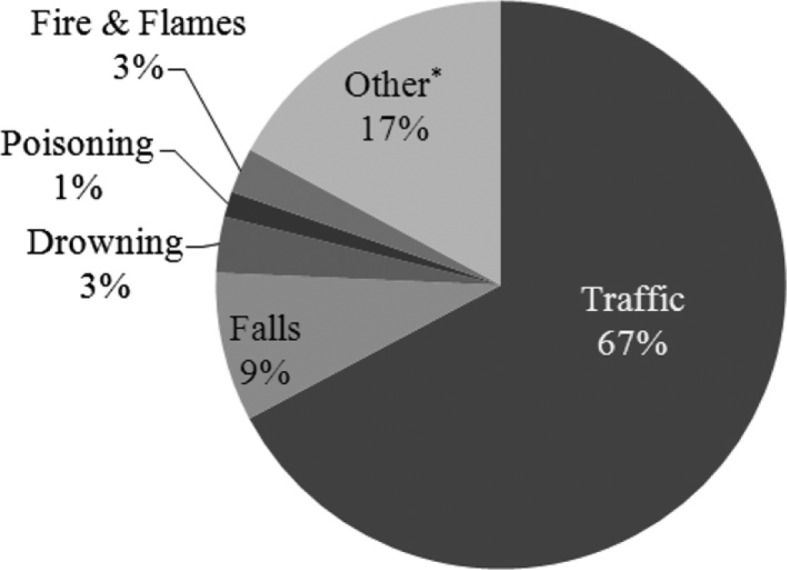
Injury deaths by external causes 2000–2008, UAE (*N*=10 079) (1). *Other refers to intentional injuries (suicide, self-inflicted, and homicide) and unintentional injuries (misadventures during medical care, accidents due to natural and environmental factors [e.g. venomous animals and plants, injury caused by animals], suffocation and foreign bodies, and other accidents [e.g. electrocution, falling objects, machinery and equipment, explosions]).

Injury is an important cause of morbidity, disability, mortality, and economic loss in the UAE and was the second leading cause of death for all age groups, with an average of 1,120 deaths per year, between 2000 and 2008 (11–15). The main circumstances contributing to death from injury were traffic-related injuries, followed by falls and drowning (Fig. 1) (1). Alarmingly, injury was the leading cause of death for children under 15 years of age and accounted for 9% of mortality between 2000 and 2008 (16). An average of 104 children died each year during 2000–2008, with an incidence rate (IR) of 13.6 per 100,000 (11–15). The predominant cause of death for this age group was traffic injury (62%; IR 13.6), followed by drowning (11%; IR 1.5) and falls (10%; IR 1.5). More males were injured compared to females (male:female ratio=1.78:1) (1). Analysis of the 2010 dataset revealed that injury remained the second leading cause of mortality accounting for 17% of all deaths in the UAE (17). The World Health Organisation estimates the traffic death rate in the UAE as 37.1 fatalities per 100,000 inhabitants per year, which is several times higher than the equivalent rates in developed Western countries, such as Australia (7.8 per 100,000 population) and the United Kingdom (5.4 per 100,000 population) (18). Recent estimates from the emirate of Abu Dhabi report that occupational injury and occupational road traffic injuries accounted for 16% of all injury deaths in 2011 (19). Consequently, occupational health has been included in the list of top public health priorities for the Health Authority of Abu Dhabi.

### Public health priority area 3: cancers

Cancer is the second leading cause of death worldwide and in all regions of the world except sub-Saharan Africa. Historically, the UAE had a much lower incidence of cancer than Western countries; however, over the last 40 years, it has undergone a period of dramatic economic, social, and demographic change, resulting in increased life expectancy and prosperity. This epidemiological transition has led to significant increases in the incidence of all chronic non-communicable diseases, including cancer, which is now the third leading cause of death in the UAE (after cardiovascular disease and injury) causing 10% of all deaths in 2010 and 16% of all deaths in the emirate of Abu Dhabi during the same year (10). At present, there is no national or regional population-based cancer registry in the UAE, which means there is no reliable information available on the incidence of cancer in the UAE. However, the Ministry of Health collected some cancer registration data for the UAE, which has been submitted for inclusion in the Globocan database (20) and Gulf Cooperation Council-wide cancer registry report, ‘Ten Year Cancer Incidence Among Nationals of the GCC States 1998–2007,’ published by the Gulf Centre for Cancer Registration (21). Data from these two sources will be used to comment on cancer in the UAE.

The Globocan data from 2008 shows that the age-standardised rates for the incidence and mortality from all cancers in the UAE is lower compared to Western countries, such as the United States (Table 2). The incidence of all cancers is projected to double by 2020, primarily due to ageing and also possibly due to increased exposure to risk factors for cancer. Breast cancer is the most common cancer among Emirati females (and the most prevalent cancer in both sexes combined), lung cancer is the most common cancer amongst Emirati males but is extremely rare in females, which reflects the prevalence of smoking (23.0% vs. 0.5%, respectively), and colorectal cancer is the second most common cancer in both sexes combined (Table 3) (20, 21). However, rates for all three types of cancer are much lower than most Western countries and are also lower than in Qatar, Bahrain, and Kuwait. This is likely to be due to the younger age of the population, less exposure to some risk factors, lower levels of screening compared to Western countries, and possibly incomplete registration. Rates for all three types of cancer are also projected to double by the year 2020 as the UAE population ages, total fertility declines, exposure time to lifestyle risk factors increases, the prevalence of obesity and diabetes increases, and due to the long latency period between starting smoking and developing lung cancer. Population-based education and awareness campaigns are urgently required to increase screening rates for at-risk individuals and to facilitate early diagnosis and prompt treatment, thereby reducing morbidity and improving survival.

**Table 2:** Summary of age standardised incidence and mortality rates from cancer in males and females in the UAE and US, 2008 (20)

	United Arab Emirates (UAE)			United States (US)		

	Male	Female	Both sexes	Male	Female	Both sexes
Population (thousands)	3,027	1,457	4,484	153,702	157,963	311,665
Number of new cancer cases (thousands)	0.8	0.8	1.6	745.2	692.0	1437.2
Age-standardised rate (W)	80.5	120.3	88.8	335.0	274.4	300.2
Risk of developing cancer before age 75 (%)	8.8	12.7	9.8	33.5	26.7	29.9
Number of cancer deaths (thousands)	0.5	0.3	0.8	294.1	271.5	565.6
Age-standardised rate (W)	56.1	60.6	54.9	121.4	90.6	104.1
Risk of dying from cancer before age 75 (%)	6.1	6.8	6.2	12.8	9.8	11.2

Age-standardised rate (W): A rate is the number of new cases or deaths per 100,000 persons per year. An age-standardised rate is the rate that a population would have, if it had a standard age structure. Standardisation is necessary when comparing several populations that differ with respect to age because age has a powerful influence on the risk of cancer. Risk of getting or dying from the disease before age 75 (%): The probability or risk of individuals developing/dying from cancer. It is expressed as the number of new born children (out of 100) who would be expected to develop/die from cancer before the age of 75, if they had cancer rates observed in the period in the absence of other causes of death (20).

**Table 3:** Five most frequent cancers by gender in the UAE and US, 2008 (20)

	United Arab Emirates (UAE)			United States (US)		

Ranking*	Male	Female	Both sexes	Male	Female	Both sexes
1	Colorectum	Breast	Breast	Prostate	Breast	Lung
2	Lung	Thyroid	Colorectum	Lung	Lung	Prostate
3	Leukaemia	Colorectum	Leukaemia	Colorectum	Colorectum	Breast
4	Non-Hodgkin lymphoma	Cervix uteri	Lung	Bladder	Corpus Uteri	Colorectum
5	Prostate	Leukaemia	Non-Hodgkin lymphoma	Non-Hodgkin lymphoma	Non-Hodgkin lymphoma	Bladder

* Ranking defined by total number of cases.

### Public health priority area 4: respiratory disorders

Respiratory illness can be acquired following exposure to gases, dusts, and fumes (as in occupational settings), from infectious agents, or as a result of poor ambient air quality. Occupational and environmental exposures can lead to obstructive (asthma, chronic bronchitis, and emphysema) or restrictive (pulmonary fibrosis) lung disease, or lung cancer. The UAE population is at a high risk of such exposures due to increased urbanisation, reliance on motorised transportation and traffic congestion, adverse weather conditions such as dust/sand storms, and the rapid expansion of the construction and manufacturing sectors emitting air-borne pollutants. Respiratory infections were the second most common non-life threatening condition in the UAE in 2010, accounting for almost 15% of all encounters across all healthcare facilities (10).

The major airports in Abu Dhabi, Dubai, and Sharjah serve as important stop-over locations for individuals travelling all over the world and receive a high-volume of travellers throughout the year. In view that contact between large numbers of people increases the risk of infection transmission, the UAE population is at potential risk from ‘new’ respiratory infections, such as ‘avian’ flu and SARS (severe acute respiratory syndrome) (22). Outbreaks of the ‘traditional’ respiratory infections, such as tuberculosis, are also possible, although this may be less likely with the national immunisation program and the visa screening requirement for all expatriate workers in the UAE.

## Recommendations

### Surveillance and monitoring

Reliable and valid longitudinal data are essential for planning population-based public health programs. As such, several areas have been identified where improvements in coordination and cooperation between different agencies of the seven emirates in the UAE will produce national datasets permitting the analysis of the magnitude and trends in occurrence of specific diseases. In addition, the establishment and continuation of several public health initiatives in the UAE should be resourced adequately. Examples include:

1. a National Cancer Registry accredited by the International Agency for Research on Cancer;
2. a cross-emirates Injury Registry and Surveillance Scheme;
3. a National Genetic Disorders Registry;
4. notification of Communicable Diseases with data from microbiology laboratories;
5. improvements in death certification and registration of births; and
6. development of laboratories to aid the recognition of specific diseases, and the analysis of environmental and/or biological samples for exposure to workplace or environmental hazards.

### Research

Research funding should be directed toward investigating the association between lifestyle and personal risk factors that are prevalent in the UAE (namely physical inactivity, unhealthy dietary practices, smoking, obesity, vitamin D deficiency, and parental consanguinity) and the development of chronic disease, such as diabetes, cardiovascular disease, and cancer. High-quality epidemiological research will provide the foundations for the development of experimental studies and clinical trials investigating the efficacy and effectiveness of various public health interventions on reducing the risk factors associated with chronic disease. Community- and school-based intervention programs focusing on increasing physical activity, improving dietary practices, increasing safety restraint use, and reducing tobacco consumption are urgently required to slow the trajectory of the estimated mortality rates due to cardiovascular disease, injury and cancer, particularly in the young population ranges.

### Training, education, and federal legislation

#### Training

Sufficient numbers of trained health professionals are required to improve the health status of the UAE. At present, there is a shortage of specialist physicians in fields, such as histopathology, oncology, occupational medicine, and infectious diseases, which hamper efforts for improving the health status of the UAE population. There are already developments in place by universities and health organisations to facilitate and improve the training of clinical and public health specialists in the UAE. The key personnel are not necessarily restricted to those directly involved in curative care or prevention. For example, engineers may be better placed to improve road safety, and reduce occupational and environmental hazards. Similarly, health education and health promotion specialists are essential for campaigns focussing on the adoption of healthy lifestyles.

#### Education

Increased focus should be placed upon improving population health through primary prevention involving health education and awareness programs. Examples include health education activities for the reduction or cessation of tobacco use, promotion of safe driving, and compliance with safe systems of work. Currently, there is a lack of workplace health and safety programs focusing on health education, safety awareness, and training. Such programs have the potential to reduce occupational-related morbidity and mortality when supported by UAE federal legislation and regulations. Finally, encouraging full uptake of immunisation is another specific health protection measure that will further enhance the health status of the UAE population.

#### Federal legislation

Legislation and standards for compliance to public health measures should be reviewed and revised periodically. Some aspects of UAE law and ministerial orders (e.g. requirements for health screening for new workers to the UAE, labour law) may benefit from cyclical reviews to ensure they are aligned with current concerns and international standards. Finally, a dual-pronged approach encompassing educational efforts coupled with federal legislation and enforcement should be adopted for certain public health issues, such as mandatory seat belt use for rear seat passengers, child safety restraints, and maximum working hours for professional drivers, to reduce the mortality attributable to traffic-related deaths.

## Conclusion

A number of public health issues, particularly non-communicable diseases, significantly contribute to morbidity, mortality, and economic losses in the UAE. In view of the population demographics of the UAE, future national population-based public health initiatives should consider the sociocultural, religious, ethnic, and educational diversity of the UAE in the design, development, and implementation of campaigns, interventions, and strategies. The major public health challenges posed by certain personal, lifestyle, occupational, and environmental factors associated with the development of chronic diseases are not isolated to the UAE; rather, they form part of a global health problem, which requires international collaboration and action.
